# Translating a “Stand Up and Move More” intervention by state aging units to older adults in underserved communities: Protocol for a randomized controlled trial

**DOI:** 10.1097/MD.0000000000016272

**Published:** 2019-07-05

**Authors:** Kevin M. Crombie, Brianna N. Leitzelar, Neda E. Almassi, Jane E. Mahoney, Kelli F. Koltyn

**Affiliations:** a Department of Kinesiology, University of Wisconsin-Madison; b Department of Medicine, University of Wisconsin School of Medicine and Public Health, Madison, Wisconsin.

**Keywords:** intervention, older adults, physical function, sedentary behavior, sitting

## Abstract

**Introduction:**

As aging is associated with functional decline, preventing functional limitations and maintaining independence throughout later life has emerged as an important public health goal. Research indicates that sedentary behavior (prolonged sitting) is associated with functional loss and diminished ability to carry out activities of daily living. Despite many efforts to increase physical activity, which can be effective in countering functional loss, only an estimated 8% of older adults meet national physical activity guidelines. Thus, shifting the focus to reducing sitting time is emerging as a potential new intervention strategy but little research has been conducted in this area. With community support and funding, we developed and pilot tested a 4-week “Stand Up and Move More” intervention and found decreases in sedentary behavior, increases in physical activity, and improvements in mobility and vitality in a small sample of older adults. The purpose of this project is to expand upon these pilot results and examine the effectiveness and feasibility of translating a “Stand Up and Move More” intervention by State Aging Units to older adults in underserved communities. Eighty older adults from 4 counties across Wisconsin predominantly made up of rural older adults and older African American adults are randomly assigned to intervention (n = 40) or wait-list control (n = 40) groups. The intervention consists of 4 weekly sessions plus a refresher session at 8 weeks, and is delivered by community partners in each county. The sessions are designed to elicit ideas from older adults regarding how they can reduce their sitting time, help them set practical goals, develop action plans to reach their goals, and refine their plans across sessions to promote behavior change. Sedentary behavior, physical activity levels, functional performance, and health-related quality of life are assessed before and after the intervention to examine the effectiveness of the program. Feasibility of implementing the program by our community partners is assessed via semi-structured interviews. Strengths of this project include strong community collaborations and a high need given that the older adult population is projected to increase substantially in the next 15 years.

**Conclusion:**

This project will provide an important step in developing effective strategies for maintaining independence in older adults through determining the feasibility and impact of a community-based intervention to break up sitting time.

## 1 Introduction

The number of adults ≥65 years in the United State is expected to more than double from 40 million in 2010 to 87 million by 2050.^[1]^ With the aging of the population, a growing number of older adults face complex health issues that often lead to functional limitations. Preservation of functional performance in community-dwelling older adults is critical to maintaining independence and quality of life, as well as containing costs in the older adult population.^[2]^ Since the elderly represent the fastest growing segment of our population, maintaining independence throughout later life has emerged as an important public health goal.^[3–5]^ Interventions that remediate functional decline are of high priority.^[6]^ Exercise has been effective in combating functional decline but older adults tend to engage in low levels of exercise, and few report meeting current national guidelines (i.e., 150 minutes of moderate intensity physical activity/wk) which may be challenging, especially for older adults with existing limitations. There is a clear need for interventions that bridge the gap between inactivity and current exercise guidelines providing older adults with an attainable transition to a more active lifestyle.

A growing body of research demonstrates the negative health consequences of sedentary behavior (i.e., too much sitting). Older adults spend approximately 60% to 70% of their waking hours engaging in sedentary activities which can increase their risk for functional decline, chronic disease development, and premature mortality.^[7]^ Although limited, research with older adults suggests that breaking up sedentary time is positively associated with improved physical function.^[8]^ Thus, interventions aimed at breaking up extended sedentary behavior by standing up and moving more throughout the day may have important benefits for older adults. However, research examining the efficacy of such interventions is scarce. With funding from the Greater Wisconsin Agency on Aging Resources (GWAAR), we recently developed a behavior change intervention designed to help older adults break up extended sitting by standing up multiple times throughout the day. Preliminary examination of the single arm pilot of the intervention, in collaboration with our community partner (i.e., the Rock County Council on Aging), indicated the intervention was feasible for staff to implement, participants expressed high satisfaction with the program, and sedentary time was reduced following participation in the intervention.^[9]^ These results suggested that our community-based behavior change intervention might effectively reduce sedentary behavior. Therefore, the purpose of the research described in this protocol is to expand upon these initial promising results and examine the effectiveness as well as the feasibility of delivery of a “Stand Up and Move More” intervention by State Aging Units to older adults in underserved communities in the state. It is hypothesized that there will be significant reductions in the primary outcome of sedentary behavior and increases/improvements in the secondary outcomes of physical activity, functional performance, and health related quality of life in the intervention group compared with no significant changes in the wait-list control group. In addition, it is hypothesized that the intervention to reduce sedentary behavior will be feasible to implement by State Aging Units to older adults in underserved communities.

## 2 Methods

### 2.1 Previous research

Our initial community partners who were instrumental in the design of the intervention included GWAAR and the Rock County Council on Aging. GWAAR provides community-based aging services to 70 counties and 11 tribes and considers interventions to increase physical activity a high priority area. Therefore, with funding from GWAAR, research was conducted to develop an intervention to reduce sedentary behavior and examine the feasibility of implementing the intervention by a State Aging Unit in a community setting. The research was conducted in 2 small urban and rural communities in Rock County, Wisconsin. The older adults in these communities had high levels of sedentary behavior (averaging almost 11 hours of their waking hours per day sitting) and their function scores were below the population average. A small-group behavior change intervention designed to break up extended sitting time by standing up multiple times during the day reduced sedentary behavior (60 min/d), was associated with moderate increases in light intensity physical activity (Cohen *d* = 0.52; 35 min/d), and large effect size improvements in mobility (i.e., gait speed; Cohen *d* = 0.74) as well as vitality (Cohen *d* = 1.15) immediately after the workshop.^[9]^ Importantly, a follow-up assessment revealed that reductions in sedentary behavior were sustained 4 weeks after the intervention ended. Strategies used most often included standing up during television commercials, and spreading household chores out across the day. Additional strategies included getting up to get a drink of water, and setting a timer to stand up. Twenty participants (out of a total of 25) completed the 4-week program and 90% of the participants expressed high satisfaction with it (e.g., “What a great program. It made me realize how much I sit during the day and helped me manage my sitting time”). Also, participants indicated that breaking up sitting time was more appealing to them than increasing exercise. Further, the intervention was found to be feasible for staff to implement. The director of the Rock County Council on Aging, who led the workshops, stated “The intervention worked because it was simple. It is not complex and that is the beauty of it.” Thus, our pilot results are quite promising and represent the development, preliminary feasibility, and piloting stages of the “Stand Up and Move More” intervention in a small sample of older adults.

As a result of these findings, the Community Research Associate with the Community-Academic Aging Research Network (CAARN; an organization that works with research faculty and counties in Wisconsin to facilitate research collaboration) put out a call to identify community partners who would be interested in administering and testing the intervention in their county. Community partners from Rock, Iowa, Vilas, and Dane Counties expressed high interest in the project and were included as the 4 study sites. There is high need in these counties. For example, Vilas County currently ranks 56 out of 72 while Rock County ranks 62 out of 72 in county health rankings. The directors of the Aging Units in these counties see the “Stand Up and Move More” intervention as a potentially viable intervention for older adults in their counties with compromised health and physical limitations. While Iowa County ranks higher in the county health rankings (29 out of 72), they have identified a lack of access to physical activity programs as a significant health risk factor. In addition, there are significant health disparities in Dane County. For example, African Americans in Dane County have higher rates of cancer, diabetes, disability, and physical inactivity than any other racial/ethnic group (HealthyDane.org). Therefore, we are including the older African American community from Madison, WI in the proposed project. An African American Program Manager has been hired to recruit and facilitate the project in the local community. As recommended by African American community leaders, a focus group of older African American adults was first conducted to ensure the intervention is culturally relevant and sensitive to the needs of the community. An experienced member of the UW Survey Center conducted a focus group of 8 to 10 older African American adults. Results were used to market and tailor the intervention to older African American adults. An African American facilitator was hired and trained to deliver the intervention.

### 2.2 Study design

**Figure 1:**
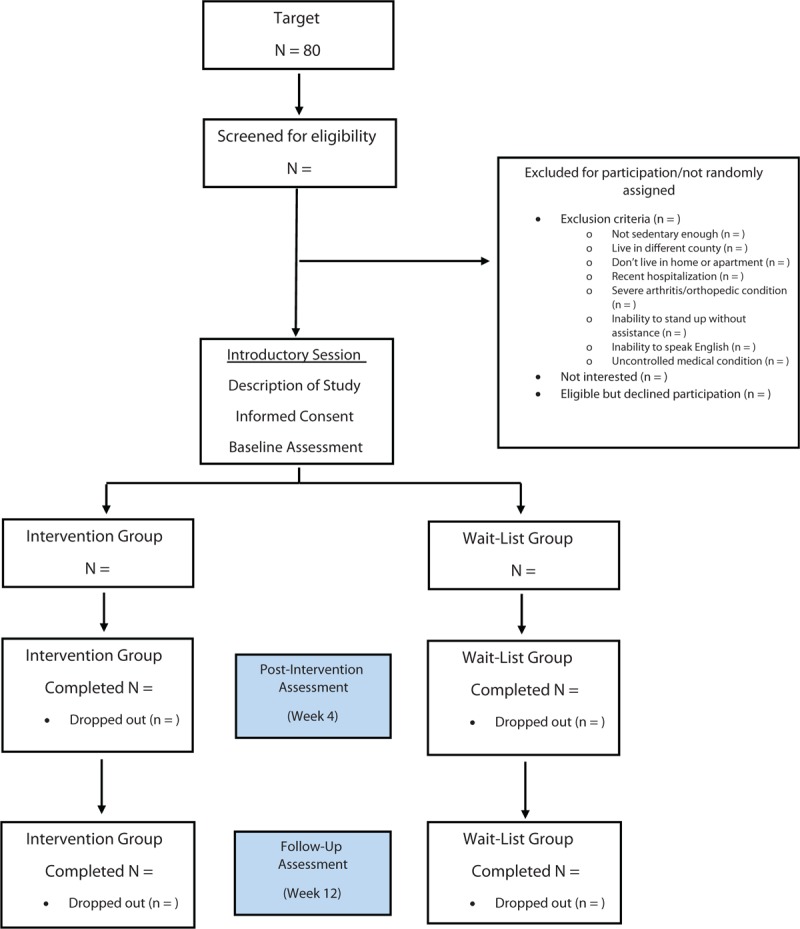
Participant flow chart.

This study is a multisite randomized controlled clinical trial designed to examine the effectiveness and feasibility of delivery of a sedentary behavior intervention by State Aging Units to underserved communities in the state. Older adults in each community are randomized into intervention or wait-list control groups (see Fig. 1). Our pilot data suggests an intervention design of one session per week for 4 weeks with a refresher session at 8 weeks is feasible for older adults to complete. The primary outcome measure is time spent engaging in sedentary behavior while the secondary outcome measures include physical activity, functional performance, and health-related quality of life. Assessments are completed at baseline (before the intervention begins), after the intervention (4 weeks after start of intervention), and at follow-up (12 weeks after the start of the intervention). The follow-up assessment time point was suggested by our community partners as being the longest reasonable time for the older adults in the control group to wait for the next workshop to begin. Feasibility of implementing the intervention is being assessed via semi-structured interviews of the health program facilitators, while feasibility of completing the “Stand Up and Move More” program by older adults is assessed by program adherence and participant satisfaction. Fidelity of delivery is being assessed by the Community Research Associate with CAARN who will travel to each site to observe a session, document fidelity, and provide feedback to each of the community partners leading the workshops.

This study is approved by the Social and Behavioral Science Institutional Review Board at the University of Wisconsin—Madison, and is registered on ClinicalTrials.gov (Identifier: NCT03412084). The UW-Madison is the IRB of record and our community partners (i.e., Aging Unit directors and health program facilitators) received human subjects training through UW-Madison.

### 2.3 Participants

A power analysis (G∗Power 3.1) was performed to estimate optimal sample size for detecting potential differences between intervention and wait-list control groups in sedentary behavior using a repeated measures design, with an alpha of 0.05, a power of 0.80, and a medium effect (0.50) based off our pilot results. Results from the power analysis indicated a total of 68 participants (34 per group) are needed for the proposed study but we have increased sample size based on our preliminary research in anticipation of a potential attrition rate of approximately 20% (a minimum of 80 adults ≥55 years will be recruited). Four counties (Rock, Iowa, Vilas, Dane) are each recruiting between 20 and 30 older adults who are randomized to either the intervention group (n = 10–15) or the control group (n = 10–15). Staff from State Aging Units in the counties are assisting with the recruitment of older adults. Recruitment strategies include announcements in senior newsletters, at congregate dining sites, churches, radio ads, and medical clinics. In addition, announcements are posted on Aging Unit websites, and flyers are sent to mailing lists of older adults in the various counties.

Individuals who are interested in learning more and potentially participating in the study are instructed to call a designated phone number (UW-Madison Exercise Psychology Lab) and leave a message. A trained research assistant promptly (within 48 hours) calls the prospective participant back and gives a general explanation of the study, asks if the individual is still interested in participating in the study, and inquires whether they are willing to complete a brief interview (i.e., phone screen) to determine eligibility. If the individual agrees to the interview, they are screened for the inclusion/exclusion criteria. In order to participate in the study, individuals must meet the following inclusion criteria: 55 years or older at time of enrollment; current residents of Rock, Iowa, Vilas, or Dane counties; and reside in a home or an apartment. Exclusion criteria are low levels of self-reported sedentary behavior (i.e., ≤6 h/d), recent hospitalization (i.e., past month), uncontrolled medical conditions (e.g., hypertension, heart disease, cancer), severe arthritis or any orthopedic condition that could be made worse by standing up and moving more, inability to stand up without assistance of another person, and inability to speak or hear spoken English. Those individuals who are deemed eligible to participate in the study are informed of an introductory meeting (i.e., date/time and location).

### 2.4 Introductory meeting and informed consent

An introductory meeting is held in each county for older adults interested in participating in the study. Community partners arrange the meeting at a location convenient for participants in their county. The principal investigator (PI) and research staff are in attendance at the meeting and a description of the study is given (i.e., to inform prospective participants of the procedures, risks and benefits, and that they are free to discontinue participation in the study at any point). The consent process takes place at the introductory meeting. Prospective participants are provided ample time to read the informed consent form and they have the opportunity to ask and obtain an adequate reply to any questions they may have. Interested and eligible participants then sign an IRB-approved consent form. Consent is obtained by qualified personnel affiliated with the study including the PI and/or research assistants. All participants receive a copy of their signed consent form. Baseline assessments (see Section 2.7) are completed after participants sign the informed consent document. In order to maintain confidentiality, participants are assigned a study ID number upon enrollment into the study that is used in place of the participant's name on all recording forms. The only list matching the study ID number to the participant's name is kept locked in the office of the PI. All computer data entry is completed using study ID numbers only. Information will not be released without written permission of the participant, except as necessary for monitoring by the IRB and National Institute on Aging (NIA). Participants are given a gift card up to $50 upon study completion ($20 for participating in the study plus $10 per each of three assessments).

### 2.5 Intervention to reduce sedentary behavior

The intervention is based on self-regulation theory as self-regulation has been shown to be an important influence of physical activity behavior change.^[10,11]^ Self-regulation has been defined as a goal-guidance process aimed at the attainment and maintenance of personal goals.^[12]^ Successful strategies for behavior change reflect a recognition that people move toward the goals they set by a process of self-regulatory actions.^[13]^ Most of the self-regulation models include components essential to the process of self-regulation such as goals about what people are trying to accomplish, self-monitoring of personal behavior and how it links up to goals, feedback and information about progress toward each goal, self-evaluation of progress, and corrective behavior leading to more effective movement toward goals.^[13]^ In addition, social cognitive theory is incorporated into the intervention due to a theoretical link between self-regulation and self-efficacy. For example, Annesi et al^[14]^ reported that use of self-regulation strategies was related to increases in self-efficacy for eating healthy and being more physically active in middle-aged adults. The current study examines whether the same is true for older adults with regards to sedentary behavior. Strategies incorporated into the “Stand Up and Move More” intervention sessions include individual goal setting, development of action plans to meet goals, information dissemination, self-monitoring, small group discussions and feedback, and various problem-solving activities. During the intervention, participants are taught how to appropriately set and adjust goals, as well as self-monitor their activity (using a small click counter every time they stand up) and completing daily logs at home at the end of each day. Older adults are asked to break up prolonged sitting (≥1 hour) with short breaks (e.g., get up and move for a couple of minutes multiple times throughout the day). Following the format of our successful pilot, participants are asked, initially, to break up sitting time an extra 3 to 5 times/d progressing to 10 to 12 times/d by the end of the 4 weeks intervention. During the first session, participants strategize on ways they can break up their sitting time safely in their home environment. In subsequent sessions, participants share strategies that worked for them and are also introduced to a list of other strategies used by Gardiner et al^[15]^ (e.g., standing up and getting a drink of water). This provides participants with a wide range of strategies and participants choose which strategies to adopt across the 4 weeks.

To support maintenance of behavior change, concepts linked to maintenance of health behaviors are integrated into the sessions (e.g., building relationships, autonomy, self-efficacy). A refresher session is held at 8 weeks after the initiation of the workshop in which the facilitator encourages participants to re-examine their motives for standing and moving more, discuss their progress and strategies which have been effective, and barriers which have prevented them from standing up and moving more throughout the day. In addition, participants revisit their goals with a focus on the future and long-term maintenance.

The intervention is being delivered via small group workshops. Research indicates the ideal range to facilitate active group discussions is 10 to 15 participants,^[16]^ thus, recruitment involves 10 to 15 older adults per workshop in order to create an environment with enough people to accumulate diverse experiences that can be shared and yet few enough to ensure that all participants can be involved in the discussions. This small group format worked well in our previous pilot research. Sessions last between 1 and 1½ hours and are in locations readily accessible to older adults to enhance participation and retention. Transportation is provided to those older adults who require transport to and from the site. To enhance reach into the older adult communities in each county, the sessions are facilitated by our community partners who regularly offer health promotion programs to older adults. During a day long training session for the “Stand Up and Move More” project led by a Community Research Associate with CAARN, the leaders are trained to act more as facilitators than as lecturers. For example, rather than prescribing specific behavior changes, they assist participants in making choices and achieving success in reaching self-selected goals. Instruction manuals have been developed with scripts provided for each session to maximize fidelity of delivery of sessions per our intervention protocol. The health program facilitators record any deviations from protocol which are discussed via weekly telephone calls with the PI. In addition, treatment fidelity monitoring (i.e., site visits) is being completed by our community research associate who has previously performed fidelity monitoring for other state health promotion programs.

### 2.6 Wait-list control group

Older adults assigned to the control group will go about their daily routines during the first 12 weeks of the study. They complete, in the same manner as the intervention group, assessments at baseline, 4, and 12 weeks later. The control participants receive the “Stand Up and Move More” intervention after 12 weeks.

### 2.7 Measures

Data collection sessions are held the week prior to the start of the intervention (at the introductory session), following the delivery of the intervention (4 weeks after the start of the intervention) and at follow-up (i.e., 12 weeks after the start of the intervention). See Table 1 for schedule of assessments.

**Table 1:**
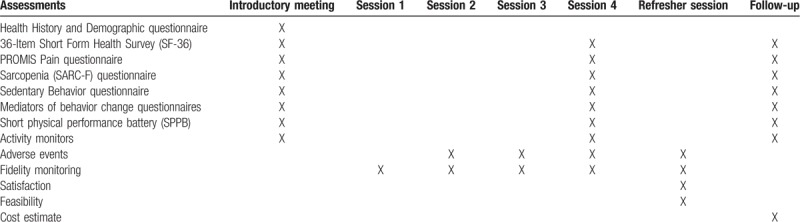
Schedule of assessments.

#### 2.7.1 Objectively measured and self-reported sedentary behavior

The primary outcome of interest is objectively measured sedentary behavior obtained via accelerometers (ActiGraph) and inclinometers (i.e., activPAL). Participants wear activity monitors at each assessment time point for 1 week. The activPAL (PAL Technologies Ltd., Glascow, UK) is a small accelerometer with an inclinometer to measure horizontal/vertical position of the device, and thus posture. This device is affixed directly to the midline of the thigh of the participant with a temporary hypoallergenic adhesive. We are using this device for the purpose of measuring time spent in sedentary behavior (mins/d) and to quantify the number of times sedentary behavior is disrupted by standing up. This device has proven to have excellent validity for the detection of posture.^[17]^ The ActiGraph (WGT3X-BT; ActiGraph, LLC, Fort Walton Beach, FL) is another small accelerometer to be worn on the hip. This device records the frequency of accelerations during ambulatory activities and can quantify physical activity across the entire spectrum (sedentary time, light intensity, moderate intensity, and vigorous intensity physical activity). We used both monitors in our preliminary research and participants found them to be acceptable to wear.^[9]^ Participants are also asked to complete a monitor wear log sheet each day. Additionally, at each assessment point, participants are interviewed by the research staff about their time spent sitting during the day in different activities (e.g., watching TV, using the computer/internet, reading) over the past week using a sedentary behavior questionnaire that has been previously validated for older adults.^[15]^

#### 2.7.2 The short physical performance battery (SPPB)

Physical function is assessed with the SPPB, which has been shown to be reliable and sensitive to change.^[18]^ The test consists of a balance test, a 4-m walk for usual gait speed, and a timed measure of chair stands.

#### 2.7.3 Health history and demographics questionnaire

At baseline, participants are asked to complete a questionnaire regarding health status and demographic information (i.e., age, sex, race, ethnicity, education, income, occupational status, and marital status), current physical activity behaviors, smoking status, current alcohol intake, and present or past history of various health conditions (e.g., cancer, diabetes, arthritis, stroke).

#### 2.7.4 The MOS 36-item short form health survey (SF-36)

The SF-36 is a widely used measure of health-related quality of life and has been shown to be reliable and valid.^[19]^ The SF-36 consists of subscales, including: general health, vitality, social functioning, mental health, physical functioning, bodily pain, and physical and emotional role functioning.

#### 2.7.5 SARC-F

The presence or absence of sarcopenia (i.e., age-related loss of muscle function) is being assessed with the 5-item SARC-F questionnaire. The SARC-F measures the cardinal features or consequences of sarcopenia, and has been shown to be reliable and valid for detecting persons at risk for adverse outcomes from sarcopenia.^[20]^

#### 2.7.6 Patient-reported outcomes measurement information system (PROMIS)

Pain is being assessed with the PROMIS pain intensity-short form and the pain interference-short form. These instruments have been shown to be reliable and valid for use with older adults.^[21]^

#### 2.7.7 Mediators of behavior change

Participants are asked to complete questionnaires assessing self-regulation strategies,^[22]^ self-efficacy,^[23]^ outcome expectancies,^[24]^ and habit strength.^[25]^

### 2.8 Analyses

Sedentary behavior outcome variables include total sedentary time (average min/d), time spent sitting in long bouts (i.e., ≥60 minutes), and average number of sit-to-stand transitions per day as measured by the activPAL. Three to 4 days of monitor wear time has been shown to capture about 80% of the inter-individual differences in activity levels of healthy adults. Thus, inclusion criteria for analyzing activity monitor data is a minimum of 10 hours per day of wear time, on at least 4 days. In addition, specific domains of sedentary behavior (e.g., watching TV, using the computer/internet, reading) are being assessed from the sedentary behavior questionnaire. A 2 (groups: intervention, wait-list control) × 3 (trials: pre-intervention, post-intervention, follow-up) repeated measures analysis of variance (ANOVA) is being used to examine differences between groups in changes in sedentary behavior before and following the intervention and control conditions. Similarly, for the secondary variables of physical activity, functional performance, health-related quality of life, and mediators of behavior change, a series of 2 × 3 repeated measures ANOVA's are being performed. Significant main effects and interactions are examined with planned contrasts. Effect sizes are calculated to examine the magnitude of differences between groups as well as the magnitude of change from pre- to post-intervention according to methods described by Cohen.^[26]^ In addition, correlational analyses are performed to examine the relationship between sedentary behavior and the secondary outcomes. Significance is set at *P* < .05. It is important to note that sample size was estimated for our primary outcome variable of sedentary behavior, and therefore, we recognize the potential limitation of our sample for secondary outcomes. However, if one or more of the secondary outcomes shows a trend in the direction of significance and/or shows a moderate to large effect size, the number of participants can be expanded in subsequent research to address a specific hypothesis.

### 2.9 Feasibility

The feasibility of implementing the program by our community partners is being assessed via semi-structured interviews. The community partners (i.e., Aging Unit directors and health program facilitators) are interviewed and asked about their satisfaction with the intervention, the ease and difficulty of implementing the program, and their likelihood of sustaining the intervention outside of a research study. (Note: Receiving feedback from our community partners is an important component of this study as Aging Units offer evidence-based health promotion programs to older adults in their counties through funding under the Older Americans Act [OAA]. When a new health promotion program is shown to be evidence-based [i.e., effective, translatable], Aging Units can use OAA funding to support and sustain implementation of the program). Interviews are transcribed and coded, classified, and organized into main themes using thematic analyses. Feasibility of completing the program by older adults is assessed on the basis of program adherence (i.e., attending at least 50% of the sessions) and participant satisfaction (brief questionnaire).

### 2.10 Cost estimate

The cost of delivering the intervention in each county is calculated based on the following: cost of training the health facilitators, payment of health facilitators for leading the workshops, cost of program materials, cost of transporting participants to the workshop sites (to ensure that older adults without transportation will be able to participate), and other miscellaneous costs incurred during the workshops (e.g., room rental for leader training).

### 2.11 Potential problems and alternative strategies

We have considered the challenge of recruiting 80 older adults into this study. We believe we will be able to recruit the sample size (i.e., 10–15 older adults per group in each county) needed for this project based on the following: there was high interest from older adults in our preliminary research, we have established good working relationships with our community collaborators who have had substantial input into the design of the current project, there are good working relationships between county Aging Units and the older adults they serve in their counties, and we will provide transport for older adults who require transportation to the site. Each county is providing 2 workshops (1 for the intervention group and 1 for the wait-list control group) over a 2-year period. If we are not able to reach our recruitment target in year 1 (recruit 75% of sample, n = 60) we will offer additional workshops or expand the program to other counties in the state in year 2.

### 2.12 Intervention safety

This intervention poses minimal risk of falling or injury. Participants are asked to think of things they can do safely in the home to break up prolonged sitting time and strategize ways to get up safely. During the weekly meetings, participants report on any situation that arose during their efforts to break up their sitting time, and then problem solve regarding alternative strategies. The PI or research assistant calls the leaders in each county on a weekly basis to discuss safety issues.

### 2.13 Data and safety monitoring

This study is overseen by a Data and Safety Monitoring Board (DSMB) which is independent from the sponsor and competing interests. The DSMB is composed of 3 members and includes representatives of the fields of relevant clinical expertise, clinical trials methodology, and biostatistics. The DSMB acts in an advisory capacity to the NIA Director to monitor participant safety, data quality, and evaluate the progress of the study. Adverse events are not expected to occur, however, the occurrence of adverse events is being assessed on an ongoing basis throughout the duration of the study (see Table 1). If patterns of recurrent adverse events across participants suggest modifications of the study protocol are required, such changes will be implemented in consultation with National Institutes of Health (NIH) staff and the IRB of record. The PI is responsible for safety monitoring of the study.

All study data are obtained solely for research purposes of this study. Source documents for each participant's data (i.e., participant binder) are stored in a locked cabinet in the PI's laboratory. Data are entered (using study ID numbers only) by one of the research assistants into a secure data management system (i.e., REDCap). The data are stored in a password protected area with firewall protection. Only designated personnel on the project have access to the data. Accuracy of data entry by the research assistants is checked by the PI on a regular basis. The PI is responsible for all data management activities included under the study protocol and manual of operating procedures. The community partners (i.e., Aging Unit directors and health program facilitators) are not involved in collecting, storing, or analyzing the data.

### 2.14 Resource/data sharing plan

The results from this study are being disseminated to our community partners and the older adults who participate in the study. In addition, the results will be disseminated to the broader aging network in the state of Wisconsin with the help of CAARN, an arm of the Wisconsin Institute for Healthy Aging (WIHA) whose mission is to promote evidence-based programs. All of CAARN's research studies are highlighted at WIHA's annual Healthy Aging Summit, where program information is shared with Wisconsin's Aging Network and partners (including public health and other private and public entities). This allows for effective generation of interest from community partners as well as a system for taking projects through all stages of research to dissemination. Further, as our research advances with subsequent larger randomized controlled trials, we will distribute the results at national venues throughout the United States (publishing in geriatric journals, giving presentations at geriatric scientific meetings). Ultimately, the intent is to disseminate our results widely so the intervention, if shown to be effective, can be adopted by a wide range of relevant stakeholders and lead to important changes in how we promote activity in older adults across the country.

## 3 Discussion

In sum, our community partners are very supportive of the project and see great need for the “Stand Up and Move More” intervention, especially since the older adult population in these counties is projected to increase substantially in the future. For example, 1 in 3 individuals in Iowa County will be aged 60 years or older while 40% to 50% of the population in Vilas County will be over the age of 60 years by the year 2040. We believe we have an exceedingly strong partnership to be able to successfully conduct this important and promising research. The project will provide an important step in developing effective strategies for maintaining independence in older adults through determining the feasibility and impact of a community-based intervention to break up sitting time.

## Author contributions

**Conceptualization:** Jane E. Mahoney, Kelli F. Koltyn.

**Data curation:** Kevin M. Crombie, Brianna N. Leitzelar, Neda E. Almassi, Jane E. Mahoney, Kelli F. Koltyn.

**Funding acquisition:** Kelli F. Koltyn.

**Investigation:** Kelli F. Koltyn.

**Methodology:** Kelli F. Koltyn.

**Project administration:** Jane E. Mahoney, Kelli F. Koltyn.

**Supervision:** Kelli F. Koltyn.

**Writing – original draft:** Kevin M. Crombie, Kelli F. Koltyn.

**Writing – review & editing:** Kevin M. Crombie, Brianna N. Leitzelar, Neda E. Almassi, Jane E. Mahoney, Kelli F. Koltyn.
